# EUPopLink Country Report—Bulgaria

**DOI:** 10.12688/openreseurope.23503.1

**Published:** 2026-04-18

**Authors:** Petar Cholakov

**Affiliations:** 1 Bulgarian Academy of Sciences,Institute of Philosophy and Sociology, Sofia, Bulgaria

**Keywords:** populism, Euroscepticism, Bulgaria

## Abstract

The article examines the connections and intersections between populism and Euroscepticism in contemporary Bulgarian politics. The emphasis is mainly on the events of the last 10 years.

The *first part* outlines the main ideological characteristics of these political actors who are both populists and Eurosceptics. Ataka, Vazrazhdane, Velichie and MECH are populist radical right (PRR). The BSP is a centre-left party; Progressive Bulgaria (PB)—the newly created project of former President R. Radev, and ITN, a centre-right party, are characterized by valence populism.

*The next section* explores the positions on Europe and the populism Euroscepticism nexus. Ataka, Vazrazhdane and Velichie want Bulgaria out of the EU. MECH criticizes the corrupt officials in Brussels but does not stand for leaving. The BSP has blasted certain EU policies, such as the Green Deal, but is generally pro-European. Radev believes that Bulgaria should not have rushed into joining the Eurozone. He disagrees with the EU’s policy towards Ukraine and is against the EU sanctions imposed on Russia. However, he does not want Bulgaria to part ways with the EU.

*The last part* outlines the responses to Euroscepticism. The mainstream parties and formations which defend pro-European positions (e.g. GERB, PP-DB, etc.) as well as the soft Eurosceptics (Radev, the BSP, etc.) have adopted several strategies towards the PRR: adversarial, accommodating, *cordon sanitaire* and incorporation.

Although Bulgarians are very pro-European, the party system is in a deep crisis: Bulgaria remains one of the most corrupt and poor countries in the EU. The country is headed towards new snap elections on April 19, 2026. PB is leading in the polls with over 10% ahead of GERB. Negative developments in the economy can boost the support for the hard Eurosceptics in the PRR. However, this is unlikely to happen in a short run.

## Introduction

Positive attitudes ^1^ towards the EU have dominated Bulgaria since the collapse of the communist regime in 1989. This observation is valid for both party systems after 1989. The first (1989–2002) was bipolar and marked by the fierce opposition between communism (BCP/BSP ^2^) and anti-communism (SDS, ODS ^3^, etc.); the second system (from 2002 until today) is “polycentric” ( Karasimeonov 2010). In the first period of bipolar competition, not only in the democratic centre-right SDS (founded in 1989) but also in the Turkish ethnic party MRF/DPS (founded in 1990) ^4^, which was engaged in a balancing act between the Left and the Right, and even in the BSP—the successor of the former communist party—there was an understanding that the country’s membership in the EU was a national priority.

With NDSV ^5^, the project of Simeon Saxe-Coburg-Gotha (founded in 2002), the bipolar party system broke down and populism flooded the political scene. But Simeon’s party, which later joined the liberal European alliance ALDE (where DPS was also a member), was strongly pro-EU. Influential populist and Eurosceptic parties emerged right before the accession of Bulgaria to the EU in 2007, under the government of Sergei Stanishev (a coalition of BSP, NDSV and DPS). Ataka was the most prominent of the first wave of populist radical right (PPR) parties. Since then, a new crop of populist Eurosceptic formations, in which the populist radical right Vazrazhdane (Revival) currently is the most significant, has emerged.

The ground for populist, hard Eurosceptic parties in Bulgaria is not as fertile now as it was before 2022. Nowadays, most Bulgarians are not pro-Russian, as they used to be in the past, but strongly pro-EU. Positive sentiments towards the EU increased between 2017, when 51% had a positive attitude towards it and 15% negative, and 2024, when 61% had a positive attitude and only 16% negative ( Ionchev 2024). Between 2017 and 2024 the attitudes towards Russia and Putin have worsened dramatically (Ibid.). After the Russian invasion in Ukraine in 2022, the negative attitudes towards Putin are shared by about 50% of Bulgarians; 34% have negative stance towards Russia (Ibid.).

R. Zhelyzakov’s (GERB) coalition government, which consisted of GERB, BSP and ITN ^6^ with the support of DPS-NN ^7^, was elected in January 2025. It declared as its top priority Bulgaria joining the Eurozone (EZ) on January 1, 2026 (for the formations in the current Bulgarian National Assembly—BNA, see Table 1 below; for their positions on key issues, see Table 2). However, as of May 2025, 50% of Bulgarians are opposed to country adopting the single currency ( “Bulgarians remain divided …” 2025). The EZ membership debate is used as mobilization tool by populist, Eurosceptic politicians.

**Table 1.** October 27, 2024 election and the formations in the 51 ^st^ BNA.

Political Party/Coalition	MPs
GERB-UDF	66
PP-DB	36
Vazrazhdane	33
DPS-New Beginning ^14^	29
BSP-United Left	19
APS (Alliance for Rights and Freedoms) ^15^	19
ITN	17
MECH ^16^	11
Velichie ^17^	10

Source: own work.

**Table 2.** Euroscepticism, populism, support for membership in the Eurozone and military aid for Ukraine in the 51 ^st^ BNA.

Political formation	VP	LWP	SE	HE	PRR	EZ	SU
GERB-UDF	x					x	x
PP-DB ^18^	x					x	x
Vazrazhdane				x	x		
DPS-NN (Peevski)	x					x	x
BSP		x	x			x	
DPS-DPS (Dogan)						x	x
ITN	x		x				x
MECH			x		x		
Velichie				x	x		

Source: own work.

VP = Valence populists; LWP = Left-wing populists; SE = Soft Eurosceptics; HE = Hard Eurosceptics; PRR = Populist Radical Right; EZ = Bulgaria must join the Eurozone as soon as possible; SU = Support for Ukraine.

(There is no party in the Bulgarian parliament that supports sending Bulgarian soldiers to the front in Ukraine).

Zhelyzakov’s government resigned on December 11, 2025 under pressure from mass protests, supported by 82% of Bulgarians ( The government of Rosen Zhelyzakov resigned 2025). The protests were sparked by the draft state budget, but they escalated into demands to change the corrupt GERB (Borisov)/DPS-NN (Peevski) model. The protests were mainly organized by the pro-European PP-DB, but were also supported by populist, Eurosceptic parties such as Vazrazhdane, as well as by sympathizers of President Radev.

## 1. Populist parties

**Ataka**, established in 2005, is the first significant populist radical right (PRR) party to call for Bulgaria leaving the EU ( “Siderov wants …” 2016). A quintessential PRR party, Ataka combines populism and hard Euroscepticism. The main emphasis in Ataka’s ideology is the need for concrete actions against “the threat of foreign powers” such as the EU and the USA ( Todorov 2012: 10–11). Ataka has vehemently opposed the EC’s attempts to close Bulgaria’s only nuclear power plant Kozloduy. The chief difference with the more recent PRR formations is the hyper nativism of the party led by Volen Siderov: its virulent polemic has been particularly aggressive against the MRF and the Roma.

The height of support for Ataka in parliamentary elections was in 2009 when it received 396,000 votes, or 9%. No PRR since then has achieved a similar number of votes. Its absolute peak in all elections was in the second round of the presidential elections in 2006 when nearly 650,000 voters (24%) supported Siderov. Ataka, together with VMRO ^8^ and NFSB ^9^ ruled together with GERB in the third government of Boyko Borisov (2017–2021). Ataka had three MEPs in the short-lived Identity, Sovereignty Tradition group in the European Parliament (2007–2009). Its support has declined since 2020. It remains out of the parliament.

**Vazrazhdane (Revival)** is a PRR, hard Eurosceptic party, founded in 2014 by Kostadin Kostadinov. The party managed to cross the 4% electoral threshold for the first time only in November 2021. It has the third largest fraction in the current parliament with 33 MPs (See Table 1 at the end). At present, the party has three MEPs out of a total of seventeen elected from Bulgaria. It is the only Bulgarian PRR represented in the European Parliament (EP), where it is part of the far-right Europe of Sovereign Nations (ESN) group. The fact that Vazrazhdane’s MEP Stanislav Stoyanov was elected in January 2025 as the leader of the ESN party is a testament to the international connections and influence of this party. By embracing both populism and hard Euroscepticism, Kostadinov’s party managed to increase its support in recent years. Vazrazhdane has vouched to sweep out “the corrupt elite”, personified not only in the leader of GERB Boyko Borisov, and the most prominent figures in the MRF – Ahmed Dogan and Delyan Peevski, but also in the Atlanticists from the PP-DB ^10^, who failed to dismantle the GERB-DPS political model, as they promised.

**Velichie** is a PRR, hard Eurosceptic party, established by businessman Ivelin Mihailov in 2023. After successfully disputing the October 2024 election results, Velichie’s 10 MPs entered parliament in March 2025 (see Table 1 below). **MECH** ^11^, led by Radostin Vasilev, is a PRR party established in 2024. Like Velichie, MECH has so far participated in two parliaments: the 50th (June 19 –November 11, 2024) and the 51st (November 11, 2024–till present). Velichie and MECH do not have systematically elaborated programmes. Both formations are relatively new, not represented in the EP, and are not yet officially part of any European party family.

**ITN (There is Such a People)** ^12^ is a socially conservative party founded in 2020 by the showman Slavi Trifonov. The party has soft Eurosceptic messages on certain topics, but the core is a combination of populism and nationalism. ITN, along with GERB, has been described as valence populist ^13^ ( Zulianello 2024). It employs strong patriotic messages without nativist overtones. Immediately after its creation, the party gained great popularity with its vehement attacks against the corrupt ‘GERB-DPS model’. It won the early elections in July 2021 (24% of the vote, 65 MPs). After that, ITN’s support plummeted. At present, ITN has 17 MPs and 1 MEP, who is a member of the European Conservative and Reformists group.

**The BSP** is a centre-left party. Its messages emphasize distributive social justice and state intervention in the economy, higher incomes, especially pensions ( BSP 2019). The overwhelming majority of BSP voters feel nostalgia for the communist era (1946–1989). Cornelia Ninova, leader of the BSP, 2016–2024, has accused the three GERB governments (2009–2013; 2014–2016 and 2017–2021), together with the MRF, of corruption and state capture. Bizarrely, during Ninova’s era the BSP had very social conservative policies (e.g. against “the gender ideology”). Ninova has been called “Orban in a skirt” ( Dainov 2019). However, the new leader of BSP, Atanas Zafirov (2024-present), changed Ninova’s course. In 2025 BSP works together with GERB and DPS-Peevski in the cabinet of Rosen Zhelyazkov (GERB). The party has 17 MPs and 2 MEPs. The BSP is a member of the Party of European Socialists. It has experienced an electoral collapse in the last 35 years: from 2.9 million votes in the parliamentary elections of 1990 to only 184,000 in the October 2024 elections. The BSP supported **Rumen Radev**’s bid for President of Bulgaria in 2016. Radev, whose second term expires in January 2027, advocates positions characterized by valence populism and soft Euroscepticism. He resigned in the beginning of 2026 in order to participate in the preterm parliamentary elections on April 19, 2026 with his project Progressive Bulgaria (PB).

## 2. Positions on Europe and the Populism Euroscepticism Nexus

According to Vazrazhdane, Bulgaria’s membership in the EU is detrimental to Bulgarian economy, which is not ready for the common market. The opening of the European labour market for Bulgarian citizens “led to a sharp loss of the working-age population on an unprecedented scale” ( “Kostadin Kostadinov …” 2024). Vazrazhdane stands for “the preservation of the Bulgarian energy industry from the “Green Deal”, which aims to close the Maritsa-Iztok power plants and the Kozloduy NPP” ( Vazrazhdane 2024).

Following the footsteps of Ataka, Vazrazhdane, MECH and Velichie claim that the memberships in the EU and the Schengen Area make Bulgaria vulnerable to flows of migrants who take the jobs of Bulgarians and commit crimes ( Otova and Staikova 2022). The nativist populism of these parties is strongly connected to their Euroscepticism.

Vazrazhdane wants Bulgaria out of NATO and has demanded a renegotiation of the terms of membership with the EU. If this process is not successful, a referendum on leaving should be held. In a similar vein, Velichie claims Bulgaria will be better off out of the EU and NATO, but differs from Vazrazhdane in the timing. Because an immediate exit will cause damage to the Bulgarian economy, the departure should take place “after 7-8 years” ( “What happened with Bulgaria?!” 2024). MECH, unlike Vazrazhdane and Velichie, is soft Eurosceptic. The party criticizes the corrupt officials in Brussels but does not stand for Bulgaria leaving the EU (or NATO).

The BSP has criticized certain EU policies, such as the Green Deal, but is generally pro-European. In May 2025, President Radev proposed to initiate a referendum on whether Bulgaria should join the Eurozone (EZ) on January 1, 2026. Vazrazhdane, Velichie and MECH supported the referendum. The BSP, however, rejected it. During Ninova’s leadership the socialists were not against the EZ, but stated that the country was not ready to become a member. However, under the leadership of Zafirov, and as a member of the Zhelyazkov’s current government, BSP now is in favour of Bulgaria joining the EZ on January 1, 2026. ITN supported the referendum in principle—as a democratic tool—but remained in favour of joining the EZ.

Both Vazrazhdane and ITN vehemently opposed the health measures during the Covid-19 Pandemic (2020–2021), which, they claimed, were imposed by Brussels. These actions, at the time, boosted the support for these formations in Bulgaria. In 2021 the country was the least vaccinated in the EU against Covid-19, only 26% were fully inoculated ( Gomez 2021).

Vazrazhdane, Velichie, MECH and president Radev are against any form of military aid to Ukraine (see Table 2). According to them solidarity with Ukraine is imposed by the EU and the corrupt Bulgarian Atlanticists (GERB, PP-DB, etc.). This refusal to support Ukraine and sympathy for Putinism—which ridicules and attacks the values of a united Europe—can be seen as manifestation of Euroscepticism. Similarly to Ataka, Revival, Velichie and MECH are PRR parties gravitating around the Kremlin. Although they share anti-American sentiments (e.g. they are against American military bases in Bulgaria), politicians like Kostadinov have welcomed the re-election of Trump.

## 3. Responses to Euroscepticism and consequences

The mainstream parties and formations which defend pro-European positions (e.g. GERB, the former Reformist Block, PP-DB, etc.) have adopted several strategies towards the PRR. The first is *adversarial.* GERB and PP-DB, for example, have highlighted the benefits of the single market and the eurozone. Contrary to Ataka, Vazrazhdane, the BSP, etc., GERB and PP-DB have accepted the political pressure from the EU to reform the Bulgarian energy sector. They are in favour of the commitments the country has made under the Green Deal (closing coal-fired power plants, reducing pollution, etc.), and are especially in favour of braking off country’s dependence on Russian oil and gas.

The BSP and Radev are implementing an *accommodation strategy*: on the one hand, they do not want Bulgaria to leave the EU, but on the other hand, they support, for example, like Ataka, Vazrazhdane, etc., Russian projects in the Bulgarian energy sector. Borisov’s governments have applied the accommodation strategy alongside the adversarial one – they have prioritized building Russian energy projects like Balkan Stream. Because of this, GERB is accused by PP-DB, for example, of playing a double game.

Other two strategies are *cordon sanitaire* and *incorporation* ( Cholakov 2018: 116–20). The track record of the latter proves to be particularly successful in taming, domesticating and suffocating PRR actors. An example of the *cordon sanitaire* strategy is Denkov’s government (2023–2024), which was supported by GERB, PP-DB and DPS. The parliamentary majority of these three formations amended the constitution in order to reduce the powers of president Radev and for the most part isolated Vazrazhdane in the legislation. An example of incorporation was the third cabinet of Borisov (2017–2021). In it GERB ruled with the United Patriots (UP), a coalition of Ataka, VMRO and NFSB (the last two were hard populist, but soft Eurosceptic and, at the time, supportive of NATO). Because the UP shared the levers of power with the centre-right, pro-EU and EPP member GERB, they were not able to materialize their radical ideas. Seen as traitors by their constituents, Ataka, VMRO and NFSB suffered a rapid decline and were left out of the subsequent parliaments. A second example is the current government of PM Zhelyazkov in which GERB might be able to ‘repeat the same magic trick’ and neutralize the BSP and the ITN.

## Conclusion

PRR parties in Bulgaria have so far not managed to gain an upper hand in the government or the parliament. Although Bulgarians are very pro-European, the party system is in a deep crisis: Bulgaria remains one of the most corrupt and poor countries in the EU. After the resignation of Zhelyzakov’s government, the country is headed towards new snap elections on April 19, 2026.

At the end of March, 2026, the polls show that the pro-European PP-DB does not receive an electoral boost despite its role in removing Borisov and Peevski from power. Instead, Progressive Bulgaria is leading in the polls with over 10% ahead of GERB, which remain second. It is early days, but it likely that PB, which thus far lacks a strong ideology, will be a valence populist and a mild Eurosceptic. Radev’s project seems to be gaining strength and momentum at the expense of formations such as Vazrazhdane, BSP, MECH and Velichie. The factors that may boost Euroscepticism and the vote for PRR in Bulgaria are: sudden, negative developments in the economy (in 2025, however, the unemployment went down, in comparison to 2024, and was merely 3.5%, and inflation—3.5%, NSI 2025; on the other hand, in the beginning of 2026, after joining the euro, the prices are creeping up); the influence of the political change in the USA under President Trump; the cooling and deterioration of relations between the White House and the EU and, last but not least, a possible favourable development for Putin in the war in Ukraine.

## Ethics and consent

Ethical approval and consent were not required.

## Data Availability

The data for this article consists of bibliographical references, which are included in the References section.

## Footnotes

^1^
The present country report was prepared as part of the EUPopLink COST Action, see Andreadis and Vasilopoulou 2026, following the EUPopLink Theoretical Framework and guidelines, see Lorimer et al. 2026.

^2^
BSP–Bulgarian Socialist Party. The formation is the successor of the Bulgarian Communist Party (BCP) which ruled the country between 1946–1990, during the communist era.

^3^
SDS (Union of the Democratic Forces) was a coalition of centre-right, social democrats, greens, etc., united by anticommunism. United Democratic Forces (UDF) referred to the coalitions around the Union of Democratic Forces, during 1996–2009. The coalition was formed in August 1996 by the UDF, the People’s Union and the MRF. It existed in this form until February 1997, when the MRF left the coalition.

^4^
Movement for Rights and Freedoms. Almost 95% of the support for this formation comes from ethnic Turks and Roma.

^5^
National Movement for Stability and Progress is a liberal, populist party.

^6^
GERB (Citizens for European Development of Bulgaria) is a conservative, centre-right, populist party founded in 2006. ITN (There is Such a People) is a populist, conservative and nationalist formation founded in 2020.

^7^
MRF-New Beginning is a political coalition, also referred to as MRF-Peevski, formed by Delyan Peevski ahead of the October 2024 parliamentary elections. The formation was the result of a leadership dispute within the MRF between Peevski, deputy chairman of MRF, and Ahmed Dogan, honorary chairman. Dogan and his supporters within MRF split into the Alliance for Rights and Freedoms (ARF/APS), also known as MRF-Dogan.

^8^
VMRO/IMRO – an acronym derived from historical Internal Macedonian Revolutionary Organization.

^9^
NFSB – National Front for the Salvation of Bulgaria.

^10^
PP-DB is a coalition founded in 2023 by two formations: PP (We Continue the Change) and DB (Democratic Bulgaria).

^11^
MECH means ‘sword’ in Bulgarian. The name of the party is an abbreviation of ‘Морал, Единство, Чест’— ‘Morality, Unity, Honor’.

^12^

Bulgarian — ИТН (Има такъв народ).

^13^
In my view, the messages of the PP (We Continue the Change) and MRF-New Beginning, also come close to the definition of valence populism in Zulianello 2024, see Table 2.

^14^
I use DPS-Peevski and DPS-New Beginning interchangeably.

^15^
APS (АПС, Алианс за права и свободи—Alliance for Rights and Freedoms) is the name of the coalition in which A. Dogan’s fraction of the DPS participated in the October 2024 election. I use APS and DPS-Dogan interchangeably.

^16^
The fraction of MECH became defunct after March 20, 2025.

^17^
Velichie successfully disputed the election results and entered the parliament in March 2025.

^18^
Whilst DB is more ideologically constituent, the PP, in my view is a valence populist. A key message of the PP is “we will achieve left goals with right means”, see Popov 2021.
