# Supplementary material for: EUPopLink Country Report—Bulgaria
Source: Open Res Eur. 2026 Apr 18;6:125. [Version 1] doi: 10.12688/openreseurope.23503.1 (PMC13270185; doi:10.12688/openreseurope.23503.1)
Supplement: Peer review history

# Reviewer report: Reviewer response for version 1

**DOI:** 10.21956/openreseurope.25442.r73228

**Published:** 2026-06-17

**Authors:** Dragomir Stoyanov

**Affiliations:** 1 University of Sussex, Brighton, England, UK

The report provides a good overview of the current party politics in Bulgaria and can be useful both to specialists and broader audiences. The report presents all significant Eurosceptic and populist parties in the country. The structure of the report includes Introduction, Populist parties, Position on Europe and Populism/Euroscepticism nexus, Responses to Euroscepticism and Consequences and Conclusion. This structure reflects the aim of the report to present the main political actors.

 Here are some suggestions for further improvement.

 At the end of the Introduction, it would be good to have a paragraph outlining the structure and the main argument of the report.

 In the section on Populist Parties, it would be good to include a definition of populist parties, and the main characteristics of such parties. It is also worth mentioning that ITN party was part of the coalition government by Zhelyazkov. A concluding sentence transitioning to the next section would also be helpful.

 In Section 2 (Euroscepticism) Euroscepticism should be explicitly defined in reference to ongoing scholarly debates. It should draw on more academic sources. The link between Euroscepticism and populism should be supported with more evidence.

 Section 3 (Responses to Euroscepticism) presents diversity of responses to Euroscepticism. However, it is not clear whether these responses changed over time. It would thus be good present a historical overview of such change.

 The conclusion provides a good summary of the report.

 In the current form, the report is rather descriptive. Making it more analytical would strengthen the argument. More engagement with literature on Euroscepticism and populism can help with this task.

 Overall, the report provides a good overview and can be strengthened with deeper analysis of the observed connections.

Is the case presented with sufficient detail to be useful for teaching or other practitioners?

Yes

Is the work clearly and accurately presented and does it cite the current literature?

Partly

If applicable, is the statistical analysis and its interpretation appropriate?

Not applicable

Are all the source data underlying the results available to ensure full reproducibility?

Yes

Are the conclusions drawn adequately supported by the results?

Partly

Is the background of the case’s history and progression described in sufficient detail?

Yes

Reviewer Expertise:

Euroscepticism, political parties, Bulgaria

I confirm that I have read this submission and believe that I have an appropriate level of expertise to confirm that it is of an acceptable scientific standard.

# Reviewer report: Reviewer response for version 1

**DOI:** 10.21956/openreseurope.25442.r75074

**Published:** 2026-06-15

**Authors:** Ivaylo Dinev

**Affiliations:** 1 Centre for East European and International Studies (ZOiS), Berlin, Germany

The case study report on Bulgaria provides a good starting point for understanding populism and populist radical right (PRR) actors in recent years. I recommend indexing after minor revisions, as outlined below.

1. **Table 2**

The columns contain abbreviations (e.g., VP) that should be explained in a note below the table to improve readability.

1. **Electoral strength of radical right parties**

The discussion of the electoral performance of radical right parties requires revision. The statement regarding Ataka's performance in 2009—"No PRR since then has achieved a similar number of votes"—is somewhat misleading, as the subsequent discussion does not adequately address the electoral support achieved by Vazrazhdane. While Vazrazhdane did not surpass Ataka's 2009 result, it nevertheless reached 14.2% of the vote and attracted a comparable number of voters, suggesting a similar trajectory of electoral development.

 Furthermore, it should be emphasized that, together with Mech and Velichie, these three parties have achieved an unprecedented combined electoral share for populist radical right actors in Bulgaria, exceeding levels observed a decade ago. This development appears to sit uneasily alongside the report's generally positive assessment of pro-European attitudes in recent years and therefore deserves further discussion.

1. **Literature**

The report would benefit from engagement with several referenced papers.

Is the case presented with sufficient detail to be useful for teaching or other practitioners?

Yes

Is the work clearly and accurately presented and does it cite the current literature?

Partly

If applicable, is the statistical analysis and its interpretation appropriate?

Not applicable

Are all the source data underlying the results available to ensure full reproducibility?

Yes

Are the conclusions drawn adequately supported by the results?

Yes

Is the background of the case’s history and progression described in sufficient detail?

Yes

Reviewer Expertise:

Social Movements and Political Parties in Central and Eastern Europe

I confirm that I have read this submission and believe that I have an appropriate level of expertise to confirm that it is of an acceptable scientific standard.

## References

[1] : Backsliding of the left: or how Viktor Orbán’s right-wing conservative illiberalism emerged as a normative ideal in Bulgarian political discourse. *European Politics and Society* .2023;24(1) : 10.1080/23745118.2021.1956239 59-76 10.1080/23745118.2021.1956239

[2] : Bulgarian political parties and European integration: from anticommunism to Euroscepticism. *European Politics and Society* .2021;22(2) : 10.1080/23745118.2020.1729050 222-236 10.1080/23745118.2020.1729050

# Reviewer report: Reviewer response for version 1

**DOI:** 10.21956/openreseurope.25442.r75069

**Published:** 2026-06-12

**Authors:** Francesco Trupia

**Affiliations:** 1 Nicolaus Copernicus University in Torun, Torun, Kuyavian-Pomeranian Voivodeship, Poland

This article provides a concise yet detailed analysis of Bulgaria's Euroscepticism vis-à-vis the development of the national political party landscape after 1989. The author provides an overview of the ideological nature of Bulgarian Eurosceptics along the right/left axis, thereby scrutinizing different right- and left-wing parties. The analysis contributes to a better understanding of Euroscepticism in Bulgaria, particularly explaining ideological ruptures and political convergences that, if taken together, provide both political scientists and policy experts with a clearer picture. The author also illuminates the too-often overlooked nature of Bulgaria’s Euroscepticism, and how the latter is instrumentalized by political parties regarding the role of Europe and the transition to full-fledged Europeanisation – both conditions widely associated with the role of Bulgaria into the institutions of the European Union and the Euro-Atlantic framework, respectively.

 The author argues that Euroscepticism plays a pivotal role in mobilizing voters and dangerously derailing the country’s path from its post-1989 geopolitical orientation westward. Nonetheless, the article would benefit from a deeper and broader analysis of the political history of Euroscepticism in Bulgaria and the Balkan region. As the latter has been culturally understood by Moscow as its satellite space of interference, the analysis would benefit from a genealogical approach to the division between Russophiles and Russophobes in Bulgaria. In doing so, the author could move beyond the right/left axis, and - if critically juxtaposed with the history of Bulgarian nationalism - hark back to issues of the post-Ottoman era through the demise of state socialism in 1989. This long-durée perspective can facilitate the author to better explain why the socialist period and the post-1989 transition to democracy have been deeply characterized by a long-lasting form of Euroscepticism. Secondly, the author could also shed light on the implications of the too-often framed civilizational mission (e.g., West/East divide) of Bulgaria and its institutional role in the Euro-Atlantic hemisphere (e.g., EU, Nato, United Nations).

 This spatial-historical lens may eventually highlight the political position of some political parties vis-à-vis recent waves of Euroscepticism. e.g., the Bulgarian Socialist Party, the heir of the former Communist Party, after Russia's invasion of Ukraine in 2022, as well as the Movement for Freedoms and Rights, namely, the de facto Turkish political party. The author clearly anticipates the results of the last elections in late April 2026. Looking at the landslide success of “Progressive Bulgaria” led by Rumen Radev, the author could further engage with the latest political developments in the country, thereby boosting the main argument and providing the latter with new data and insights.

Is the case presented with sufficient detail to be useful for teaching or other practitioners?

Yes

Is the work clearly and accurately presented and does it cite the current literature?

Partly

If applicable, is the statistical analysis and its interpretation appropriate?

Yes

Are all the source data underlying the results available to ensure full reproducibility?

Yes

Are the conclusions drawn adequately supported by the results?

Partly

Is the background of the case’s history and progression described in sufficient detail?

Yes

Reviewer Expertise:

Political Philosophy, History of Ideas, Post-socialist and post-colonial studies, minority and democracy;

I confirm that I have read this submission and believe that I have an appropriate level of expertise to confirm that it is of an acceptable scientific standard, however I have significant reservations, as outlined above.

# Reviewer report: Reviewer response for version 1

**DOI:** 10.21956/openreseurope.25442.r73230

**Published:** 2026-05-21

**Authors:** Adriana Ștefănel

**Affiliations:** 1 Faculty of Journalism and Communication Studies, Universitatea din Bucuresti, Bucharest, Bucharest, Romania

The manuscript offers a timely and informative overview of the relationship between populism and Euroscepticism in contemporary Bulgarian politics, with a particular focus on developments over the last decade. One of the main strengths of the article lies in its detailed contextual knowledge of the Bulgarian political landscape and its attempt to map the positions of a broad range of political actors in relation to the populism–Euroscepticism nexus. The text is generally well structured, accessible, and potentially useful both for comparative research and for teaching purposes, especially for readers less familiar with Bulgarian politics.

 At the same time, several aspects of the manuscript would benefit from further clarification and strengthening in order to improve its analytical consistency and conceptual rigor.

 First, although the article provides substantial descriptive information, the analytical progression of the case remains at times underdeveloped. Several classifications, particularly those related to “valence populism”, “hard” and “soft” Euroscepticism, and the categorization of parties as populist radical right actors, would benefit from clearer theoretical justification and more explicit operationalization. At present, some of these labels appear more interpretative than systematically demonstrated.

 Second, the engagement with the broader comparative literature on populism and Euroscepticism could be expanded. While the manuscript cites relevant recent contributions connected to the EUPopLink framework, the theoretical discussion would be strengthened by a more systematic dialogue with foundational and contemporary scholarship in the field. This would help situate the Bulgarian case more clearly within wider comparative debates.

 Third, the manuscript occasionally shifts from analytical discussion toward a more journalistic or normative register. Certain formulations, particularly in the sections discussing corruption, geopolitical alignments, or future political developments, would benefit from a more neutral academic tone and more cautious wording. Similarly, some statements in the conclusion regarding future electoral dynamics and geopolitical scenarios appear somewhat speculative and are not always fully supported by the empirical material presented in the article.

 Finally, while the article functions primarily as a descriptive country report rather than a methodological research article, it would still benefit from a brief clarification of the analytical basis used for classifying parties and positions (e.g., party manifestos, public speeches, expert interpretation, secondary literature, media statements, or other sources).

 Overall, the manuscript represents a valuable contribution to the comparative study of populism and Euroscepticism in Central and Eastern Europe. The suggested revisions are intended primarily to strengthen the conceptual precision, analytical consistency, and academic framing of the article.

Is the case presented with sufficient detail to be useful for teaching or other practitioners?

Yes

Is the work clearly and accurately presented and does it cite the current literature?

Partly

If applicable, is the statistical analysis and its interpretation appropriate?

Yes

Are all the source data underlying the results available to ensure full reproducibility?

No source data required

Are the conclusions drawn adequately supported by the results?

Partly

Is the background of the case’s history and progression described in sufficient detail?

Partly

Reviewer Expertise:

NA

I confirm that I have read this submission and believe that I have an appropriate level of expertise to confirm that it is of an acceptable scientific standard.
